# Terpenoids from *Platostoma rotundifolium* (Briq.) A. J. Paton Alter the Expression of Quorum Sensing-Related Virulence Factors and the Formation of Biofilm in *Pseudomonas aeruginosa* PAO1

**DOI:** 10.3390/ijms18061270

**Published:** 2017-06-14

**Authors:** Tsiry Rasamiravaka, Jérémie Ngezahayo, Laurent Pottier, Sofia Oliveira Ribeiro, Florence Souard, Léonard Hari, Caroline Stévigny, Mondher El Jaziri, Pierre Duez

**Affiliations:** 1 Laboratory of Plant Biotechnology, Université Libre de Bruxelles, rue des Professeurs Jeener et Brachet 12, 6041 Gosselies, Belgium; jaziri@ulb.ac.be; 2 Laboratoire de Pharmacognosie, Bromatologie et Nutrition Humaine, Faculté de Pharmacie, Université Libre de Bruxelles, CP 205/09, Boulevard du Triomphe, 1050 Bruxelles, Belgium; Laurent.Pottier@ulb.ac.be (L.P.); sofia_oliveira27@hotmail.com (S.O.R.); florence.souard@ujf-grenoble.fr (F.S.); Caroline.Stevigny@ulb.ac.be (C.S.); pierre.duez@umons.ac.be (P.D.); 3 Centre de Recherche Universitaire en Pharmacopée et Médecine Traditionnelle (CRUPHAMET), Faculté des Sciences, Université du Burundi, BP 2700 Bujumbura, Burundi; lhari2000@yahoo.fr; 4 Unit of Therapeutic Chemistry and Pharmacognosy, Université de Mons (UMONS), Bâtiment VI, Chemin du Champ de Mars 25, 7000 Mons, Belgium; 5 Laboratoire de Biotechnologie et Microbiologie, Département de Biochimie Fondamentale et Appliquée, Faculté des Sciences, Université d’Antananarivo (UA), BP 906, Antananarivo 101, Madagascar; 6 Département de Pharmacochimie Moléculaire, Université Grenoble Alpes, 38000 Grenoble, France; 7 Département de Pharmacochimie Moléculaire, Centre National de Recherche Scientifique, 38000 Grenoble, France

**Keywords:** anti-virulence, biofilm, *Platostoma rotundifolium*, Lamiaceae, *Pseudomonas aeruginosa*, quorum sensing

## Abstract

*Platostoma rotundifolium* (Briq.) A. J. Paton aerial parts are widely used in Burundi traditional medicine to treat infectious diseases. In order to investigate their probable antibacterial activities, crude extracts from *P. rotundifolium* were assessed for their bactericidal and anti-virulence properties against an opportunistic bacterial model, *Pseudomonas aeruginosa* PAO1. Whereas none of the tested extracts exert bacteriostatic and/or bactericidal proprieties, the ethyl acetate and dichloromethane extracts exhibit anti-virulence properties against *Pseudomonas aeruginosa* PAO1 characterized by an alteration in quorum sensing gene expression and biofilm formation without affecting bacterial viability. Bioguided fractionation of the ethyl acetate extract led to the isolation of major anti-virulence compounds that were identified from nuclear magnetic resonance and high-resolution molecular spectroscopy spectra as cassipourol, β-sitosterol and α-amyrin. Globally, cassipourol and β-sitosterol inhibit quorum sensing-regulated and -regulatory genes expression in *las* and *rhl* systems without affecting the global regulators *gacA* and *vfr*, whereas α-amyrin had no effect on the expression of these genes. These terpenoids disrupt the formation of biofilms at concentrations down to 12.5, 50 and 50 µM for cassipourol, β-sitosterol and α-amyrin, respectively. Moreover, these terpenoids reduce the production of total exopolysaccharides and promote flagella-dependent motilities (swimming and swarming). The isolated terpenoids exert a wide range of inhibition processes, suggesting a complex mechanism of action targeting *P. aeruginosa* virulence mechanisms which support the wide anti-infectious use of this plant species in traditional Burundian medicine.

## 1. Introduction

Assuming that the success of bacterial infection relies on an optimal expression of virulence [1], an attractive anti-pathogenic approach consists in targeting these mechanisms [2,3,4]. Indeed, virulent bacteria are able to colonize their hosts by building biofilms, disseminating with different types of motility and releasing multiple virulence factors [5]. Besides, the persistence of bacterial infection is linked to their ability to form structured biofilms which represent a protective barrier against antibiotics and immune defense, allowing survival and further re-dissemination [6,7]. Interestingly, these faculties are intimately interconnected to quorum sensing (QS), a bacterial cell-to-cell communication that allows bacteria to coordinate their behavior depending on their population density, through the release and perception of small diffusible molecules called “auto-inducers” [8] which finally induce the production of several virulence factors and modulate bacterial behaviors, including biofilm lifestyle growth [9].

*P. aeruginosa* has become a model organism of studying this bacterial communication and harbors several QS mechanisms that depend on the type of the released signaling molecules [9]. The acyl homoserine lactones (AHLs) *N*-3-oxo-dodecanoyl-l-homoserine lactone (3-oxo-C12-HSL) and *N*-butanoyl-l-homoserine lactone (C4-HSL) are produced and sensed, respectively, by the LasI/R and RhlI/R QS systems. *LasR* and *rhlR* genes encode *LasR* and RhlR regulator proteins, respectively, while *lasI* and *rhlI* genes encode the LasI and RhlI synthases necessary for the synthesis of 3-oxo-C12-HSL and C4-HSL, respectively [10]. The PQS system (*Pseudomonas* quinolone signal) is based on 2-alkyl-4-quinolones [11] and requires several enzymes encoded by *pqsABCDE*, *phnAB* and *pqsH* operons and *PqsR* regulator [12]. Altogether, full activation of theses QS systems enhances the production of several virulence factors such as rhamnolipids, pyocyanin, LasB elastase, hydrogen cyanide and cytotoxic lectins [9].

Many bioactive molecules have been reported to exert modulatory properties on bacterial virulence expression and most of them are naturally-derived compounds, such as halogenated C-30 and C-56 furanones, inspired from natural compounds produced by the marine macroalga *Delisea pulchra*, which reduce biofilm and target the QS systems in *P. aeruginosa*, an important human, animal and plant pathogen [13]. In view of further investigating natural products for QS-modulating properties, African medicinal plants were investigated as they represent a largely untapped source for the discovery of bioactive compounds with novel mechanisms of action [14]. *Platostoma rotundifolium* (Briq.) A. J. Paton is widely used in traditional Burundian medicine against microbial diseases [15] and its aerial parts were shown to contain triterpenic acids (e.g., ursolic and corrosolic acids) with direct and indirect bactericidal properties against *Escherichia coli* as well as sensitive and methicillin-resistant *Staphylococcus aureus* [16], contributing to explain its purported anti-infectious properties. In the present study, we report on antibacterial activities of *P. rotundifolium* extracts towards *P. aeruginosa* PAO1, an opportunistic pathogen which particularly infects immunocompromised patients and describe the isolation, the identification and the characterization of antibacterial properties of major isolated bioactive compounds.

## 2. Results

### 2.1. Antibacterial Activities of the P. rotundifolium Extracts

**Figure 1:**
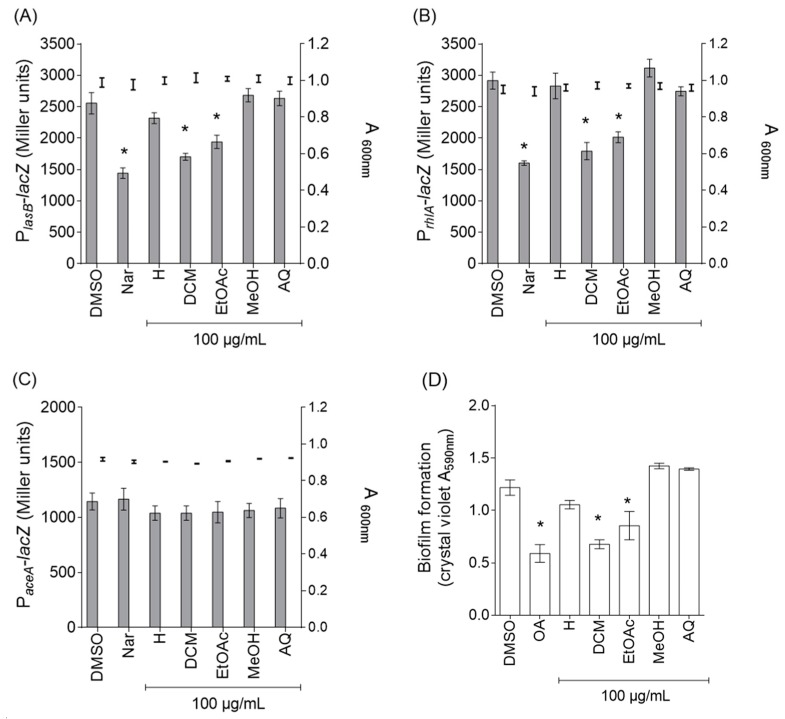
Anti-virulence effects of *Platostoma rotundifolium* extracts in *P*. *aeruginosa* PAO1: (**A**) effect of *P. rotundifolium* extracts on the expression of QS-regulated *lasB* gene; (**B**) effect of *P. rotundifolium* extracts on the expression of QS-regulated *rhlA* gene; (**C**) effect of *P. rotundifolium* extracts on the expression of QS-independent *aceA* gene; and (**D**) effect of *P. rotundifolium* extracts in biofilm formation. Extracts (H: *n*-hexane, DCM: dichloromethane, EtOAc: ethyl acetate, MeOH: methanol, AQ: aqueous) were tested at 100 µg/mL; naringenin (Nar, 4 mM) is used as a reference quorum sensing inhibitor; oleanolic acid (OA, 800 µM) is used as an anti-biofilm control and dimethylsulfoxide (DMSO, 1%) as a solvent control. The cell density of the bacteria was assessed at 600 nm (bold error bars) and gene expression was measured as the β-galactosidase activity of the *lacZ* gene fusions and expressed in Miller units (grey bar). Biofilm formation was quantified by crystal violet staining and measured as A_590nm_. Error bars represent the standard errors of the means; all experiments were performed in quintuplicate with three independent assays and asterisks indicate samples that are significantly different from the DMSO (One-way ANOVA followed by Dunnett’s test of multiple comparisons; *p* < 0.01).

The MIC (Minimum Inhibitory Concentration) and MBC (Minimum Bactericidal Concentration) of five *P. rotundifolium* aerial part extracts with different degrees of polarity were higher than 4000 µg/mL on *P. aeruginosa* PAO1, indicating an extremely weak potential as a direct antibiotic; by comparison, the MIC and MBC for tobramycin, an antibiotic generally used to treat patients with cystic fibrosis infected by *P. aeruginosa* [17], were 1 and 2 µg/mL, respectively (data not shown). To investigate anti-virulence property, the effects of these *P. rotundifolium* extracts on the expression of two virulence factors genes (*lasB*, encoding for the virulence factor LasB elastase, and *rhlA*, encoding for the precursor of the virulence factor rhamnolipids, both genes are regulated by QS system in *P. aeruginosa*) as well as on biofilm formation by *P. aeruginosa* PAO1 were assessed. The flavanone naringenin known as virulence factor gene inhibitors [18] and the triterpenoid oleanolic acid known to reduce biofilm formation in *P. aeruginosa* [19] were used as positive controls. As shown in Figure 1, the ethyl acetate (EtOAc) and dichloromethane (DCM) extracts at 100 μg/mL inhibit the expression of *lasB* (34 ± 3% and 30 ± 6% of inhibition, respectively; Figure 1A) and *rhlA* (30 ± 2% and 35 ± 3% of inhibition, respectively; Figure 1B) genes without affecting bacterial growth and reduce biofilm formation by *P. aeruginosa* PAO1 (35 ± 3% and 43 ± 7% of inhibition, respectively; Figure 1D). Besides, these extracts had no effect on a QS-independent gene (isocitrate lyase-encoding *aceA* gene; Figure 1C), suggesting that the observed effects on QS genes expression is specific and does not result from a global inhibition of *P. aeruginosa* PAO1 metabolic activity [20]. Hexane, methanol (MeOH) and aqueous (AQ) extracts have no effect either QS-dependent (*lasB* and *rhlA*) genes expression nor on biofilm formation (Figure 1). To conclude, *P. rotundifolium* extracts exhibit a potential antibacterial property that is not associated with bacteriostatic and/or bactericidal activity but rather with anti-QS and anti-biofilm activities in *P. aeruginosa* model.

Since both EtOAc and DCM extracts showed practically similar TLC chromatographic profiles with higher intensity spot area for EtOAc extracts (data not shown), this latter was subjected to further chromatographic fractionations.

### 2.2. Fractionation of the EtOAc Extract and Isolation of Major Bioactive Compounds

**Figure 2:**
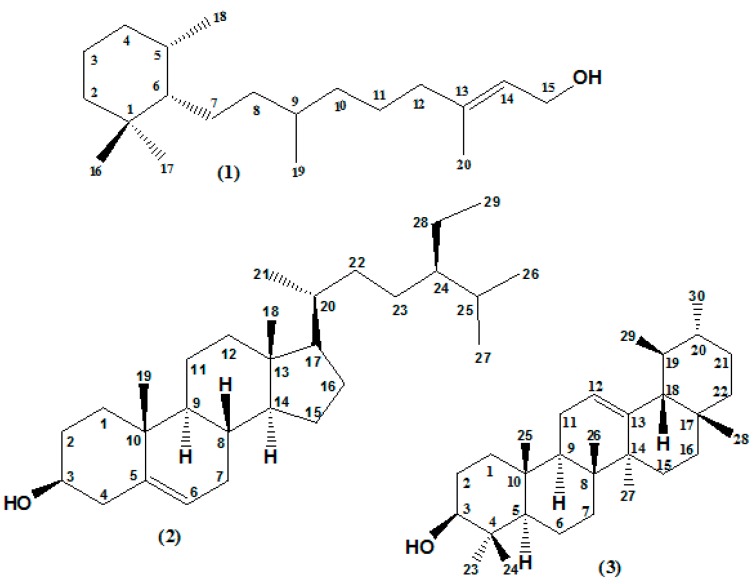
Structures of isolated bioactive terpenoids from *P. rotundifolium*. Cassipourol, β-sitosterol and α-amyrin (compounds **1**–**3**, respectively).

*P. rotundifolium* EtOAc extract was fractionated by column chromatography using a gradient mixture of DCM and EtOAc with increasing polarities (10% to 100% of EtOAc). Fractions were monitored by TLC and pooled (according to their chromatographic profiles) to give eight fractions (F1–F8), which were then evaluated for their potential effects on QS-regulated *lasB* and *rhlA* gene as well as on biofilm formation. Fractions F1–F4 were all active in inhibiting *lasB* and *rhlA* gene expression, whereas only F2 and F4 inhibit biofilm formation (Figure S1). F2 fraction, the most active fraction (Figure S1) was then submitted to flash chromatography and twelve sub-fractions were collected and were further subjected to preparative TLC for the isolation of the major compounds, yielding compounds **1** (11 mg) from sub-fraction F2(7), **2** (10 mg) from sub-fraction F2(8) and **3** (7 mg) from sub-fraction F2(4) (Figure 2). The structures of isolated compounds were elucidated using NMR (1D and 2D) and high-resolution (HR) MS experiments, comparing with literature data. These compounds were identified as cassipourol (**1**) [21], β-sitosterol (**2**) [22,23] and α-amyrin (**3**) [24,25,26] (Tables S2 and S3, Figure 2).

### 2.3. Isolated Compounds Exert Anti-QS and/or Anti-Biofilm Properties on P. aeruginosa PAO1

**Figure 3:**
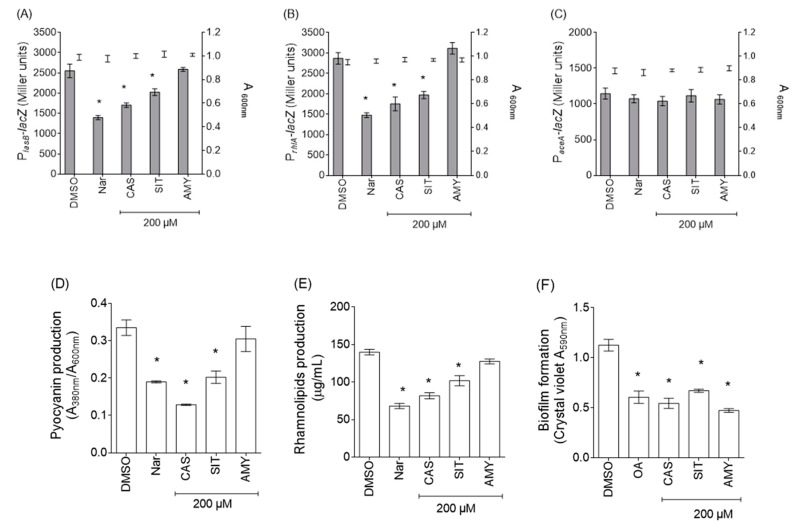
Anti-virulence effects of isolated compounds from *P. rotundifolium* EtOAc extracts in *P*. *aeruginosa* PAO1: (**A**) effect of isolated compounds on the expression of QS-regulated *lasB* gene; (**B**) effect of isolated compounds on the expression of QS-regulated *rhlA* gene; (**C**) effect of isolated compounds on the expression of QS-independent *aceA* gene; (**D**) effect of isolated compounds in biofilm formation; (**E**) effect of isolated compounds on pyocyanin production; and (**F**) effect of isolated compounds on rhamnolipids production. Each isolated compound was tested at 200 µM; naringenin (Nar, 4 mM) is used as a reference QS inhibitor; oleanolic acid (OA, 800 µM) is used as an anti-biofilm control and dimethylsulfoxide (DMSO, 1%) as a solvent control. The cell density of the bacteria was assessed at 600 nm (bold error bars) and gene expression was measured as the β-galactosidase activity of the *lacZ* gene fusions and expressed in Miller units (grey bar). Biofilm formation was quantified by crystal violet staining and measured at A_590nm_. Pyocyanin was extracted, quantified by absorbance measurements at 380 nm and calculated as the ratio between A_380nm_ and A_600nm_. The rhamnolipids production was measured using methylene-blue-based method and expressed in µg/mL. Error bars represent the standard errors of the means; all experiments were performed in quintuplicate with three independent assays and asterisks indicate samples that are significantly different from the DMSO (One-way ANOVA followed by Dunnett’s test of multiple comparisons; *p* < 0.01). AMY: α-amyrin; CAS: cassipourol; and SIT: β-sitosterol.

**Figure 4:**
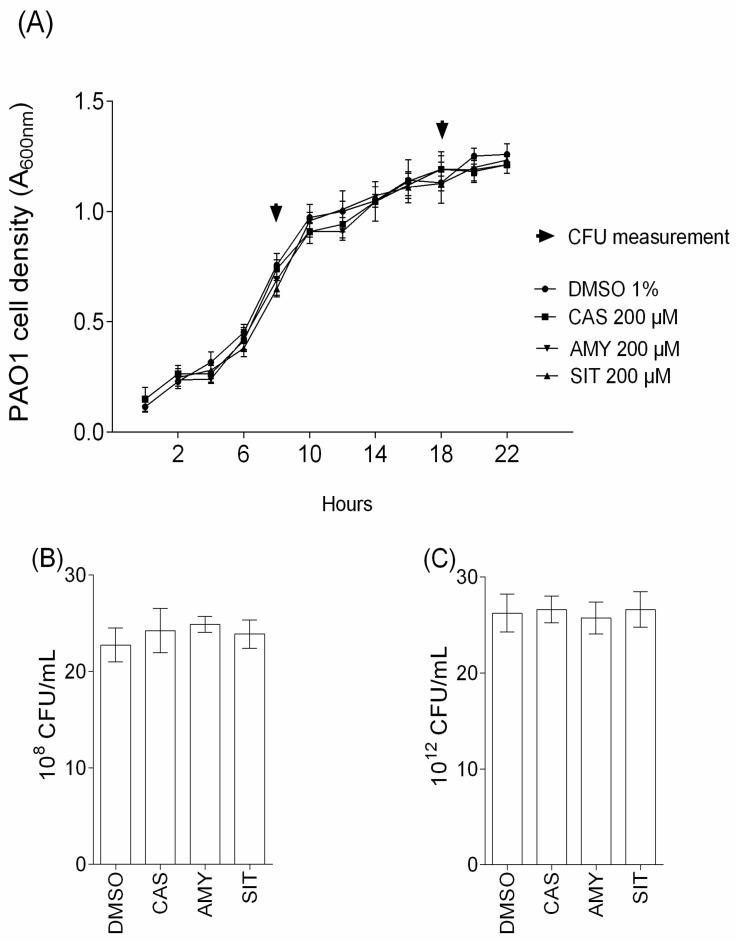
Effect of cassipourol, β-sitosterol and α-amyrin on the growth and viability of *P. aeruginosa* PAO1. (**A**) Growth kinetics of PAO1 in presence of cassipourol or β-sitosterol or α-amyrin at 200 µM or DMSO 1% over a period of 22 h. The cell density of the bacteria was assessed as A_600nm_ and colony forming units (CFU) were quantified after: 6 h (**B**); and 18 h (**C**). The statistical significance of each test (*n* = 3) was evaluated by conducting One-way ANOVA followed by Dunnett’s test of multiple comparisons (i.e., each test was compared with the control condition, DMSO), and a *p* value of <0.01 was considered as significant.

Cassipourol, β-sitosterol and α-amyrin were investigated for their effects on QS-regulated *lasB* and *rhlA* genes expression, QS-related virulence factors production and biofilm formation in *P. aeruginosa* PAO1. Isolated compounds were tested at 200 µM, an arbitrary concentration previously used by Rasamiravaka et al. [4] to investigate anti-virulence activities of terpenoids compounds. As shown in Figure 3, cassipourol and β-sitosterol, at 200 µM, significantly reduce the expression of *lasB* (37 ± 4% and 25 ± 5% of inhibition, respectively; Figure 3A) and *rhlA* (46 ± 4% and 32 ± 5% of inhibition, respectively; Figure 3B), without affecting *P. aeruginosa* growth and the expression of the QS-independent gene *aceA* (Figure 3C), whereas α-amyrin had no effect on the expression of these genes. Besides, when *P. aeruginosa* PAO1 was grown in the presence of cassipourol or β-sitosterol, a drastic inhibition of the production of pyocyanin (75 ± 4% and 44 ± 3% of inhibition, respectively; Figure 3D) and rhamnolipids (45 ± 4% and 34 ± 3% of inhibition, respectively; Figure 3E) was recorded. This effect was not observed in presence of α-amyrin. Interestingly, Cassipourol, β-sitosterol and α-amyrin inhibited biofilm formation (52 ± 4%, 44 ± 3% and 55 ± 2%, respectively; Figure 3F). To ensure that the decrease in biofilm biomass and virulence factors production is not due to an inhibition of growth, growth kinetics and CFU measurements were recorded in the presence of cassipourol, β-sitosterol or α-amyrin at 200 µM. As shown by turbidity (Figure 4A) and CFU (Figure 4B,C) measurements, the cell growth and viability of *P. aeruginosa* PAO1 were not affected by any of these compounds over all stages of bacterial growth.

### 2.4. Cassipourol and β-Sitosterol Affect the Expression of lasI/R, rhlI/R in P. aeruginosa PAO1 but Not the Global Activator Genes gacA and vfr

**Figure 5:**
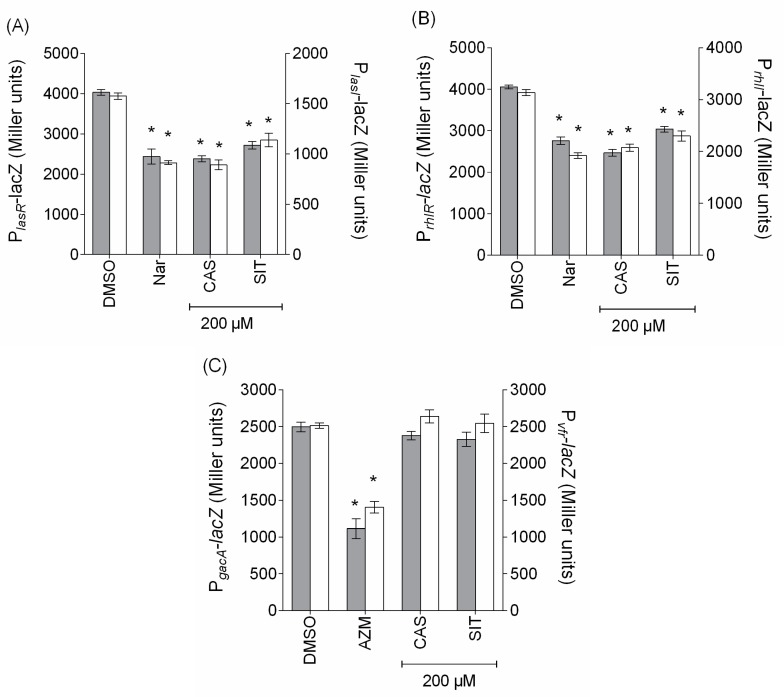
Effect of cassipourol and β-sitosterol on QS genes (*lasI/R* and *rhlI/R*) and global activator genes (*gacA* and *vfr*) expression in *P. aeruginosa* PAO1: (**A**) effect of cassipourol, β-sitosterol and α-amyrin on *lasR* (grey bar) and *lasI* (clear bar) expression following 18 h of growth; (**B**) effect of cassipourol, β-sitosterol and α-amyrin on *rhlR* (grey bar) and *rhlI* (clear bar) expression following 18 h of growth; and (**C**) effect of cassipourol, β-sitosterol and α-amyrin on *gacA* (grey bar) and *vfr* (clear bar) expression following 18 h of growth. Each isolated compound was tested at 200 µM. Naringenin (Nar, 4 mM) or Azithromycin (AZM, 2 µg/mL = 2 µM) are used as a quorum sensing inhibitor control. Gene expression was measured as the β-galactosidase activity of the *lacZ* gene fusions and expressed in Miller units. Error bars represent the standard errors of the means; all experiments were performed in quintuplicate with three independent assays and asterisks indicate samples that are significantly different from the DMSO (One-way ANOVA followed by Dunnett’s test of multiple comparisons; *p* < 0.01).

As cassipourol and β-sitosterol impact on QS-dependent *rhlA* and *lasB* gene expression, we evaluated the impact of both compounds on QS-regulatory *lasI/R* and *rhlI/R* genes that positively control the expression of *rhlA* and *lasB* genes [27]. Similarly, we evaluated their impact on the global activators *gacA* and *vfr* that exert positive effects on the transcriptional regulators *LasR* and RhlR [28,29]. Cassipourol and β-sitosterol at 200 µM significantly reduce the expression of the *lasI* and *lasR* genes (50 ± 3% and 43 ± 5% of inhibition, respectively; Figure 5A) and of the *rhlI* and *rhlR* genes (44 ± 3% and 39 ± 3% of inhibition, respectively; Figure 5B) but not of the global activator genes *gacA* and *vfr* (Figure 5C). This suggests that both compounds impair QS at the level of the *las* and *rhl* systems circuitry in *P. aeruginosa* PAO1, which consequently reduce the production of pyocyanin (Figure 3D) and rhamnolipids (Figure 3E).

### 2.5. Isolated Terpenoids Disrupt the P. aeruginosa PAO1 Biofilm Formation in a Dose-Dependent Manner

**Figure 6:**
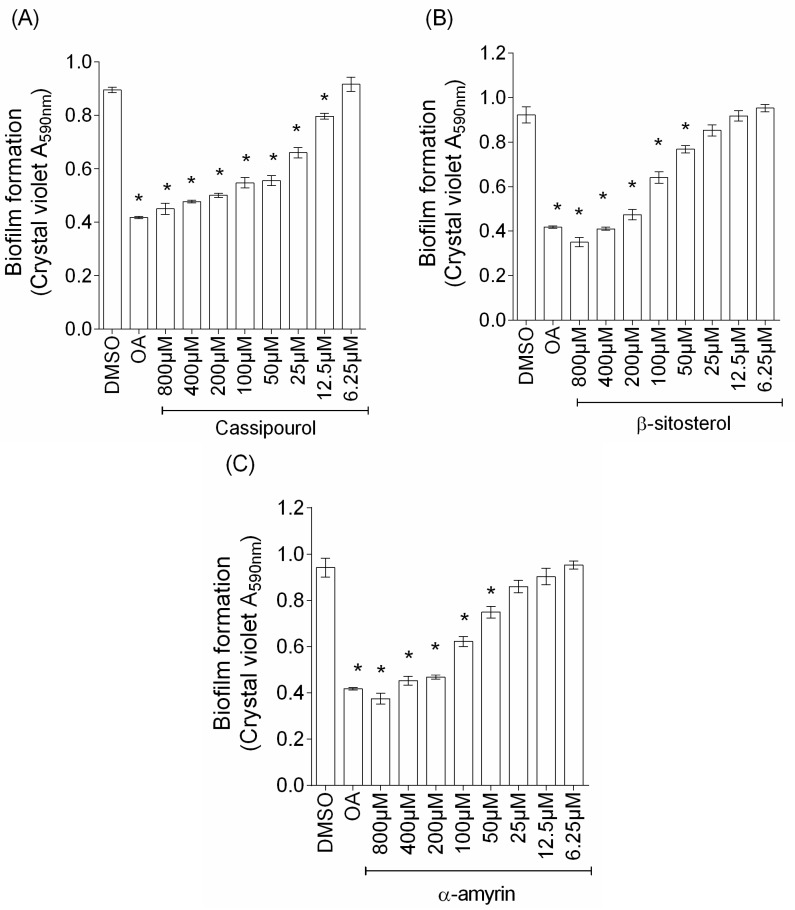
Dose-dependent anti-biofilm activity of: cassipourol (**A**); β-sitosterol (**B**); and α-amyrin (**C**). The biofilm formation of *P. aeruginosa* PAO1 grown in minimal medium supplemented with DMSO 1%, oleanolic acid 800 μM (OA) or different concentrations of purified compounds (from 6.25 to 800 µM) after incubation without agitation at 37 °C for 24 h. Biofilm formation was quantified by crystal violet staining and measured as A_590nm_. All experiments were performed in triplicate with three independent assays. Error bars represent the standard errors of the means; all experiments were performed in quintuplicate with three independent assays and asterisks indicate samples that are significantly different from the DMSO (One-way ANOVA followed by Dunnett’s test of multiple comparisons; *p* < 0.01).

As three compounds inhibit biofilm formation, we further characterize this anti-biofilm property by primarily investigating their effects in a dose-dependent manner. As a result, anti-biofilm activity is greatly enhanced by increasing the concentrations of tested compounds (Figure 6), suggesting a dose-dependent activity for cassipourol (in the range 12.5–800 µM; IC_50_ of 180 µM), β-sitosterol (50–800 µM; IC_50_ of 200) and α-amyrin (50–800 µM; IC_50_ of 190). Additionally, in the testing for synergies by checkerboard method, combinations of active compounds reveal indifferent FIC indexes (FICI cassipourol (100 µM)/α-amyrin (100 µM) = 1; FICI cassipourol (100 µM)/β-sitosterol (100 µM) = 1; FICI β-sitosterol (100 µM)/α-amyrin (100 µM) = 1).

### 2.6. Isolated Terpenoids Disrupt the P. aeruginosa PAO1 Biofilm Phenotype

**Figure 7:**
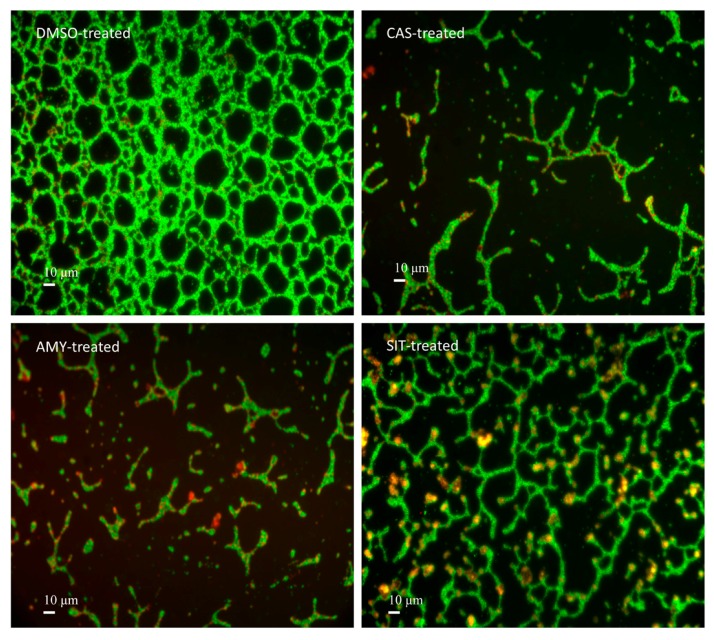
*P. aeruginosa* PAO1 biofilm phenotypes as affected by cassipourol or β-sitosterol or α-amyrin. *P. aeruginosa* PAO1 cells were incubated statically at 37 °C for 24 h for biofilm formation in presence of DMSO 1%, or cassipourol (CAS) or β-sitosterol (SIT) or α-amyrin (AMY) at 100 µM. Cells were visualized after staining with SYTO-9 (green fluorescence for living bacteria) and propidium iodide (red fluorescence for dead bacteria) furnished in the LIVE/DEAD *Bac*Light kit. Fluorescence microscopy was achieved by using a Leica DM IRE2 inverted fluorescence microscope using a 40× objective lens and images were false-colored and assembled using Adobe Photoshop.

**Figure 8:**
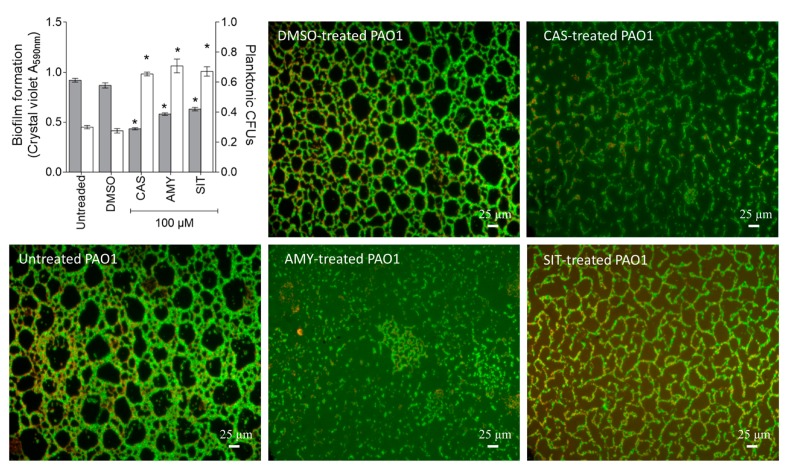
Effect of cassipourol, β-sitosterol and α-amyrin on a one-day-old preformed biofilm by *P. aeruginosa* PAO1. *P. aeruginosa* PAO1 cells were incubated for 24 h and then treated or not for 24 h with DMSO 1%, or cassipourol (CAS) or β-sitosterol (SIT) or α-amyrin (AMY) at 100 µM. Biofilm phenotypes were visualized as in Figure 6. Additionally, biofilm formation was quantified by crystal violet staining and measured as A_590nm_ and CFU measurement of planktonic bacteria and biofilm-encapsulated bacteria treated for 24 h with DMSO 1% or cassipourol (CAS) or β-sitosterol (SIT) or α-amyrin (AMY) at 100 µM on a one-day-old culture. Quantification of biofilm formation by *P. aeruginosa* grown in in minimal media (grey bar) and CFU measurement (clear bar) of planktonic bacteria after static incubation at 37 °C for 24 h. Biofilm formation was quantified by crystal violet staining and measured at A_590nm_ and planktonic bacteria by CFU measurement. Error bars represent the standard errors of the means; all experiments were performed in quintuplicate with three independent assays and asterisks indicate samples that are significantly different from the DMSO (One-way ANOVA followed by Dunnett's test of multiple comparisons; *p* < 0.01).

Fluorescence microscopy indicated that *P. aeruginosa* PAO1 grown for 24 h in static control condition forms a thick and homogenous biofilm layer on coverslips with good cell-to-cell connections interspaced by uncolonized surfaces (Figure 7; DMSO). By contrast, cassipourol-, β-sitosterol-, α-amyrin-treated *P. aeruginosa* PAO1 cells failed to establish compact cell-to-cell attachment resulting in a reduction of microcolonies confluence (Figure 7). As the three compounds impair the formation of *P. aeruginosa* PAO1 biofilms, we further examined theirs impact on pre-formed *P. aeruginosa* PAO1 biofilm. As shown in Figure 8, the addition of cassipourol, β-sitosterol and α-amyrin to one-day-old pre-formed biofilms results in a loss of compact and heterogeneous structures, leading to biofilms mainly composed by isolated bacterial clumps and disorganized microcolonies structure. This remarkable modification is confirmed by a quantitative reduction of biofilm as measured by crystal violet method (for cassipourol-, β-sitosterol and α-amyrin, 47 ± 3%, 34 ± 3% and 38 ± 2% of reduction, respectively; Figure 8). Conversely, a two-fold increase of planktonic bacteria population is recorded for treated cultures by CFU quantification (Figure 8), suggesting that these compounds induce bacterial dispersion out of a pre-formed biofilm.

### 2.7. Isolated Terpenoids Exhibit Synergistic Activity with Tobramycin against Biofilm-Encapsulated P. aeruginosa PAO1

**Figure 9:**
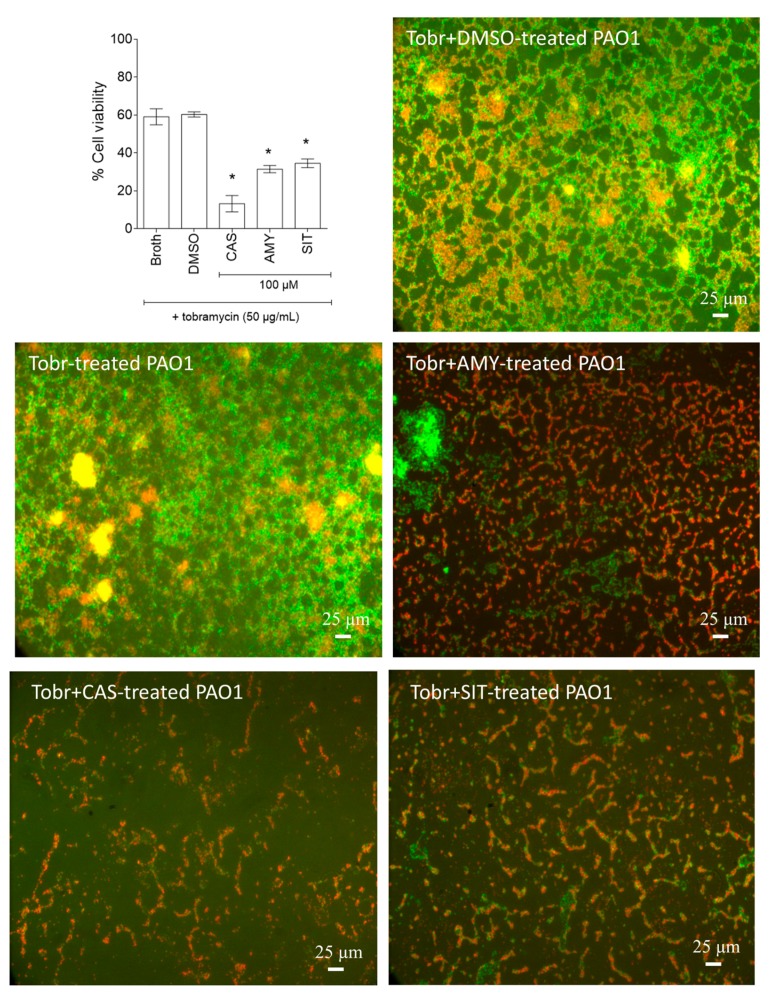
Synergistic activity of cassipourol, β-sitosterol and α-amyrin with tobramycin against biofilm-encapsulated *P. aeruginosa* PAO1. *P. aeruginosa* PAO1 cells were incubated statically for 24 h and then treated for 24 h with tobramycin (50 μg/mL = 107 µM) alone or with DMSO 1% or cassipourol (100 μM) or β-sitosterol (100 µM) or α-amyrin (100 µM). Assessment of bacterial viability and microscopy were performed as in Figure 6 and Figure 7. Error bars represent the standard errors of the means; all experiments were performed in quintuplicate with three independent assays and asterisks indicate samples that are significantly different from the DMSO (One-way ANOVA followed by Dunnett’s test of multiple comparisons; *p* < 0.01).

Given the disruption of biofilm structure induced by the isolated *P. rotundifolium* terpenoids, we hypothesized that biofilm-encapsulated bacteria would become more accessible to an antibiotic treatment. Accordingly, the effectiveness of tobramycin combined with purified compounds was evaluated on one-day-old biofilms. The addition of cassipourol, β-sitosterol or α-amyrin (100 µM) considerably improved the effectiveness of tobramycin (50 μg/mL = 107 µM) against *P. aeruginosa* PA01 (Figure 9) with a drastic reduction in cell viability of biofilm-encapsulated bacteria (89 ± 2%, 69 ± 2% and 71 ± 2%, respectively versus 40 ± 5% for DMSO treatment). As such effects may arise from a simple synergistic effect between tested compounds and tobramycin, thus we evaluated the FIC index of terpenoid-tobramycin combinations in *P. aeruginosa* planktonic stage by checkerboard method, in the concentrations range 6.25–800 µM. Results show that there is no synergistic effect but rather indifference (FICI tobramycin/α-amyrin = 1; FICI tobramycin/β-sitosterol = 1; FICI tobramycin/α-amyrin = 1), suggesting that the increased effectiveness of tobramycin in biofilm-encapsulated bacteria is not associated with an increase of the intrinsic antibiotic activity of tobramycin.

### 2.8. Isolated Terpenoids Affect P. aeruginosa PAO1 Swimming and Swarming but Not Twitching Motilities

**Figure 10:**
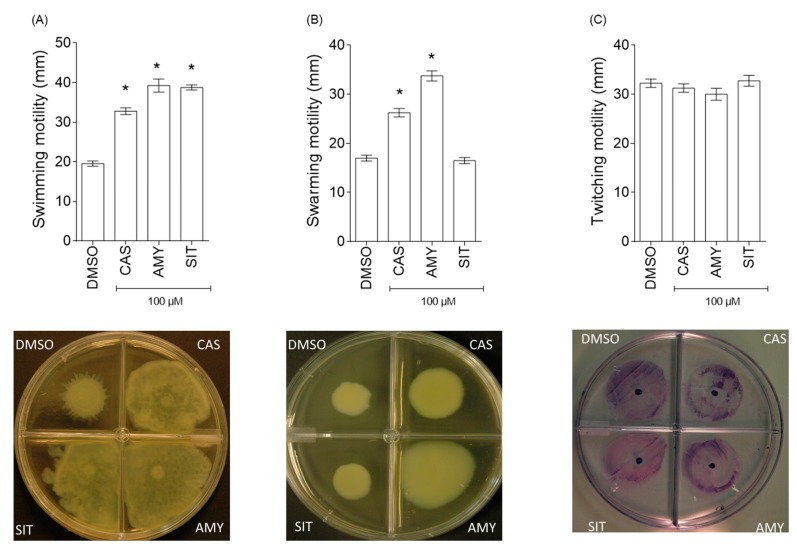
Effect of cassipourol, β-sitosterol and α-amyrin on *P. aeruginosa* PAO1 motilities. (**A**) Swimming motility of *P. aeruginosa* PAO1 onto LB agar (0.3%) supplemented with DMSO (1%) or cassipourol (CAS, 100 μM), β-sitosterol (SIT, 100 μM) or α-amyrin (AMY, 100 μM); (**B**) Swarming motility of *P. aeruginosa* PAO1 onto LB agar (0.6%) supplemented with glutamate (0.05%), glucose (0.2%) and DMSO (1%) or cassipourol (CAS, 100 μM), β-sitosterol (SIT, 100 μM) or α-amyrin (AMY, 100 μM). After incubation at 37 °C for 24 h, the zones of migration (down) from the point of inoculation were measured (up) for each condition; and (**C**) Twitching motility of *P. aeruginosa* PAO1 onto LB agar (1%) supplemented with DMSO (1%) or cassipourol (CAS, 100 μM), β-sitosterol (SIT, 100 μM) or α-amyrin (AMY, 100 μM). The twitching zones were stained (down) and their diameters (up) measured after incubation at 37 °C for 48 h. Error bars represent the standard errors of the means and all experiments were performed in quintuplicate with three independent assays and asterisks indicate samples that are significantly different from the DMSO (One-way ANOVA followed by Dunnett’s test of multiple comparisons; *p* < 0.01).

Figure 10 shows that motilities are differently affected by the three isolated terpenoids. Although the swimming motility is promoted in presence of cassipourol (84 ± 4% promotion), β-sitosterol (110 ± 6%) and α-amyrin (104 ± 5%) (Figure 10A), the swarming motility is promoted only by cassipourol (80 ± 6%) and α-amyrin (103 ± 4%) (Figure 10B), and the twitching motility does not appear to be affected by any of the isolated terpenoids (Figure 10C). Globally, these results are correlated with the increased proportion of planktonic bacteria population in presence of these terpenoids (Figure 8).

### 2.9. Isolated Terpenoids Reduce Total Extracellular Polysaccharides

**Figure 11:**
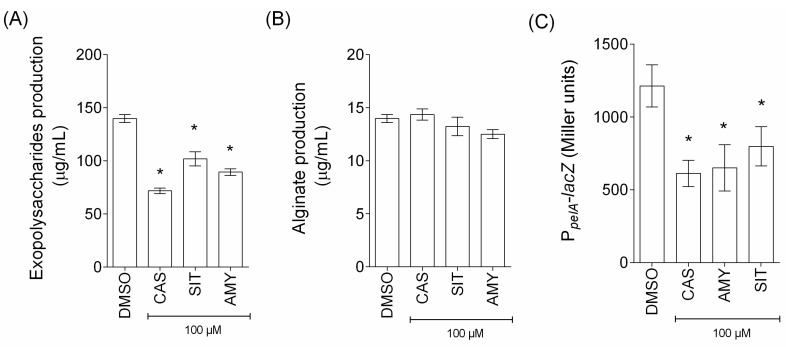
Effect of cassipourol, β-sitosterol and α-amyrin on extracellular polysaccharides production by *P. aeruginosa* PAO1. (**A**) Quantification of total extracellular polysaccharides: The cell density of the bacteria was assessed at 600 nm and extracellular polysaccharides production was measured using Phenol-Sulfuric Acid method and expressed in μg/mL with glucose as standard; (**B**) Quantification of alginate: The cell density of the bacteria was assessed at 600 nm and alginate production was measured using carbazole method and expressed in μg/mL with sodium alginate as standard; and (**C**) Effect of cassipourol, β-sitosterol and α-amyrin on the expression of *pelA* gene: Gene expression was measured as the β-galactosidase activity of the *lacZ* gene fusions and expressed in Miller units. Each compound was tested at 100 µM. Error bars represent the standard errors of the means; all experiments were performed in quintuplicate with three independent assays and asterisks indicate samples that are significantly different from the DMSO (One-way ANOVA followed by Dunnett’s test of multiple comparisons; *p* ≤ 0.01).

Microbial cells in biofilm are covered by extracellular polymeric substance mainly composed of polysaccharides. *P. aeruginosa* produces three polysaccharides (Alginate, Pel and Psl) that are determinant for the stability of the biofilm structure [30]. As shown in Figure 11A, the amounts of extracellular polysaccharides produced by *P. aeruginosa* PAO1 over 24 h growth were reduced in cassipourol-, β-sitosterol- and α-amyrin-treated *P. aeruginosa* PAO1 cells (65 ± 3%, 42 ± 3% and 30 ± 2% inhibition, respectively) whereas the production of the acidic polysaccharide alginate was not impacted (Figure 11B). Interestingly, cassipourol, β-sitosterol and α-amyrin reduced the expression of the *pelA* gene (45 ± 5%, 32 ± 7% and 40 ± 7% inhibition, respectively; Figure 11C) encoding for a protein with a predicted polysaccharide deacetylase and glycoside hydrolase domain implicated in the production of Pel, a cationic polysaccharide that crosslinks extra-cellular DNA [31,32]. This suggests that the global extracellular polysaccharides reduction is at least in part related to a decrease in Pel polysaccharides, which consequently weakens the biofilm structures of *P. aeruginosa* PAO1.

## 3. Discussion

Medicinal plants have been used for millennia to treat and appease various ailments, notably diseases mediated by pathogenic bacteria [14,15]. Recent findings indicate that, beyond their curative effectiveness through a bactericide or bacteriostatic mode of action, plant bioactive constituents can also contribute to limit the development of bacteria within infected hosts through the disruption of bacterial virulence, undermining the strength of pathogenic bacteria [33,34]. Direct and indirect bactericidal properties of *P. rotundifolium* triterpenic acids (e.g., ursolic and corrosolic acids) against Gram-positive *S. aureus* (sensitive and methicillin-resistant) and Gram-negative *E. coli* have already been described by our group [16]. The present study indicates that the aerial part of *P. rotundifolium* exert anti-virulence rather than bactericidal activities towards the Gram-negative opportunistic pathogen *P. aeruginosa*. Thus, *P. rotundifolium* produces myriad of secondary metabolites which exert bactericidal and/or anti-virulence activity against different bacteria species which may increase the probability of plant defense success against bacterial invasion.

Although this is the first report of such properties on *Platostoma* genus, other plants species in the Lamiaceae family have already shown bacterial anti-virulence properties. For instance, the aqueous extracts of *Ocimum sanctum* L. (Lamiaceae) reduce the QS-mediated production of violacein in *Chromobacterium violaceum* as well as the production of pyocyanin, staphylolytic protease, elastase and biofilm in *P. aeruginosa* PAO1 without affecting bacterial growth [35]. Likewise, five Lamiaceae ethanolic extracts (*Thymus vulgaris* L., *Ocimum basilicum* L., *Origanum vulgare* L., *Salvia officinalis* L. and *Rosmarinus officinalis* L.) inhibited QS-mediated virulence factors in *P. aeruginosa*. Thymol, a phenolic monoterpene isolated from *Thymus vulgaris* has been shown to be one of the active components [36].

Herein, the bioguided fractionation of the *P. rotundifolium* EtOAc extract led to the isolation of three non-bactericidal terpenoids that present anti-virulence properties on biofilm and QS, inhibiting the QS-dependent expression of *rhlA* and *lasB* genes and reducing the production of virulence factors, pyocyanin and rhamnolipids. Among the isolated compounds, cassipourol is isolated for the first time from the genus *Platostoma*. To the best of our knowledge, its anti-QS and anti-biofilm properties on *P*. *aeruginosa* are also reported for the first time in this work. The two other isolated compounds, β-sitosterol and α-amyrin, were known in the literature for their anti-biofilm properties on *Listeria monocytogenes* at 1 mM [37] and on *P. aeruginosa* at 75 µM [38], respectively.

According to the literature, several terpenoids have been shown to exhibit bacterial anti-virulence activities. For instance, some terpenoids inhibit: (i) the morphogenesis, adhesion, and biofilm formation in *Candida albicans* as the example of linalool and farnesol [39]; (ii) the biofilm formation and elastase production in *P. aeruginosa* and *Staphylococcus aureus* as the example of viridiflorol, ursolic and betulinic acids [40]; or (iii) the production of native auto-inducers acylhomoserine lactones in *P. aeruginosa* [41] and the QS-controlled violacein production in *C. violaceum* as the example of the sesquiterpene lactones derivatives [42].

Cassipourol and α-amyrin inhibit biofilm formation and conversely improve the two flagellar-dependent (swimming and swarming) motilities, promoting planktonic lifestyle in *P. aeruginosa* PAO1. This phenotype is mostly observed in *P. aeruginosa* with low levels of intracellular c-di-GMP where bacterial dispersion and planktonic lifestyle are promoted to the detriment of structured biofilm [43]. In addition, study conducted by Chua et al. [44] have demonstrated that the expression of the *pelA* gene is positively regulated by c-di-GMP by using construction strain harboring P_BAD_-*yhjH* vector with P_pel_-*lacZ* fusion which overproduce phosphodiesterase in presence of arabinose leading to an increase degradation of c-di-GMP. Accordingly, additional experiments that explore the c-di-GMP pool concentration in *P. aeruginosa* PAO1 treated with these compounds should be carried out in order address any implication of c-di-GMP concentration fluctuation. Intriguingly, swarming motility of *P. aeruginosa* PAO1 is not promoted in presence of β-sitosterol contrary to swimming motility. As swarming motility is also influenced by type IV pili [45] and rhamnolipids [46], it is suggested that these factors may be differently affected in presence of β-sitosterol, cassipourol and α-amyrin leading to variation in swarming motility phenotype. However, the swarming spreading in LB medium is quite difficult to appreciate; the use of another medium, such as the iron-limited medium [47] might be more adequate to appreciate the bacterial spreading and to confirm the hyper-swarming motility phenotype (Figure 10B).

In *P. aeruginosa* PAO1, Psl polysaccharide is also important for biofilm formation [48]. Indeed, Psl functions as a scaffold, holding biofilm cells together in the matrix and the *psl* operon was shown to be essential for biofilm formation in PAO1 strains as Psl is involved in early- and late-stages of biofilm development and needed for maintenance of the biofilm structure post-attachment [49]. Interestingly, Wagner [50] demonstrated through microarray analysis that gene *pslB* was QS regulated in the *P. aeruginosa* PAO1, and Gilbert et al. [51] demonstrated that *LasR* could bind to the promoter region of the *psl* operon, suggesting the link between QS and the expression of the *psl* locus. Thus, Psl polysaccharides might be at the crossroad of QS and biofilm formation intrication. Consequently, quantification of Psl polysaccharides in presence of these compounds should be carried out to detect an eventual decrease in Psl polysaccharide production. Beyond the plausible disruption of *las* and *rhl* systems, the decrease of pyocyanin production could also suggest a disruption of PQS system circuitry. Indeed, the complex formed by PQS signal molecule and PqsR are directly implicated in activation of pyocyanin production independently of *rhl* system [52]. Thus, impact of these compounds on PQS system should be carried out by using for instance biosensor-based assay proposed by Fletcher et al. [53] which will allow evaluating the production of PQS signal molecules in presence of these compounds.

Nevertheless, the biofilm structures of *P. aeruginosa* PAO1 induced by these compounds are drastically impaired, suggesting that the modulation of c-di-GMP concentration and/or the QS system circuitry may not be enough to explain the overall observed biofilm phenotype alteration. Altogether, the three isolated terpenoids exert a wide range of anti-virulence activities, suggesting complex mechanisms of action that deserve further transcriptomic analysis to better characterize their impact on *P. aeruginosa* PAO1 behavior. Interestingly, the chemical backbone of cassipourol presents structural similarities to the native auto-inducers acylhomoserine lactones (AHLs: *N*-(3-oxododecanoyl)-l-homoserine lactone and *N*-butanoyl-l-homoserine lactone; Figure S2) which bind the transcriptional regulators *LasR* and RhlR, respectively, to induce virulence factors gene expression in *P. aeruginosa* PAO1, including the *rhlA* and *lasB* genes [9]. This plausible interaction of cassipourol with the ligand-binding domains in *LasR* and RhlR should be investigated as well as its possible competition with AHLs for *LasR* and/or RhlR binding. Likewise, α-amyrin shares chemical features with ursolic acid (Figure S2) which is known to inhibit biofilm formation by *P. aeruginosa* and to promote motility without interfering with QS [40,54]. Actually, α-amyrin is the biosynthesis precursor of ursolic acid [55], which suggests that other ursane-type triterpenes may exert anti-biofilm properties with similar mechanisms of action.

Their abilities to disperse preformed biofilm and to improve the effectiveness of tobramycin towards biofilm-encapsulated *P. aeruginosa* PAO1 are encouraging for further investigation in eventual therapeutic and/or industrial application and it would be interesting to evaluate their in vivo effect in a model of infection (e.g., *Galleria mellonella*) [56]. It should be noted that: (i) cassipourol exhibits cytotoxicity with IC_50_ values of 2.4 and 2.8 μg/mL (8.2 and 9.5 µM) against the A2780 human ovarian cancer cell line [21] and larvicidal activity against *Culex quinquefasciatus* [57]; (ii) the cytotoxicity of α-amyrin has not been reported yet conversely to its anomer β-amyrin which exhibited weak cytotoxic activities against human bladder cancer cells (NTUB1) [58]; and (iii) β-sitosterol is already used and commercialized as dietary supplement without genotoxicity and cytotoxicity [59].

Cassipourol exerts a wider range of activities, at lower concentrations compared to β-sitosterol and α-amyrin, and appears as the best synthon for further structure-activity studies for developing more active, non-toxic and stable compounds which could be used to restrict the virulence of important pathogenic bacteria.

## 4. Materials and Methods

### 4.1. Plant Material and Extracts

*Platostoma rotundifolium* (Briq.) A.J. Paton aerial parts (mostly composed by leaves, stems and flowers) were harvested from the Nyabiraba area (1730 m, S 03.45325°, E 029.47607°) in Bujumbura Rural Province (Burundi). The plant was identified by the specialists of the Herbarium of the National Botanical Garden of Belgium where a voucher specimen has been deposited under the number BR0000013315900. Plant extraction was conducted as previously described [16]. Briefly, 1700 g of powdered plant material were successively extracted by 8 L of each of five solvents of increasing polarities (*n*-hexane, dichloromethane, ethyl acetate, methanol and water), yielding 16.4, 49.9, 18.4, 52.8 and 125.6 g, respectively. Plant extracts were stored at −20 °C until use and a portion of each extract was dissolved in dimethylsulfoxide (DMSO) to get appropriate concentrations for biological tests.

### 4.2. Bacterial Strains, Plasmids, and Culture Conditions

*P. aeruginosa* PAO1 strain and its derivatives (Table S1) were grown (37 °C, agitation 175 rpm) in LB-MOPS broth (50 mM, pH 7) supplemented with carbenicillin (300 µg/mL) or tetracycline (15 µg/mL) when appropriate. Plasmids (Table S1) were used and introduced in *P. aeruginosa* PAO1 as previously described [60]. PAO1/P_pelA_-lacZ strains were obtained from Singapore Centre on Environmental Life Sciences Engineering (SCELSE), Nanyang Technological University, Singapore.

### 4.3. Chemicals and Solvents

Naringenin and oleanolic acid were purchased from Sigma-Aldrich and dissolved in 100% DMSO. Antimicrobial drugs (tobramycin and azithromycin) were purchased from TCI^®^ (Tokyo chemical industry Co., LTD, Tokyo Japan) and dissolved in deionized water. Solvents were analytical grade, obtained from VWR International (Leuven, Belgium) and redistilled before use. All other chemicals were also analytical grade and purchased from Sigma Aldrich (St. Louis, MO, USA).

### 4.4. Antibacterial Assay and Assessment of Kinetic Bacterial Growth

For MIC (Minimum Inhibitory Concentration) and MBC (Minimum Bactericidal Concentration) determination, *P. aeruginosa* PAO1 was grown on 24-well microplates with 1 mL of LB broth in presence of *Platostoma rotundifolium* extracts or fractions or purified compounds at different concentrations (from 31.25 to 4000 µg/mL) and incubated at 37 °C for 24 h. The MIC was defined as the lowest antimicrobial concentration that completely inhibited growth as detected by the naked eye [61]. All inhibited growth cultures were then sub-cultured onto LB agar plate and incubated at 37 °C for 24 h to determine the MBC which was defined as the lowest concentration that yielded negative sub-cultures [62]. Tobramycin, a widely used antibiotic to treat *P. aeruginosa* lung infection in cystic fibrosis patients was selected as positive control [17]. Additionally, the effect of active compounds on *P. aeruginosa* PAO1 growth kinetic was assessed by evaluating PAO1 cell density at A_600nm_ with a SpectraMax M2 device (Molecular Devices, Silicon Valley, CA, USA) over 22 h culture, confirmed by cell counting (colony-forming units, CFU) at times 8 and 18 h.

### 4.5. Gene Expression and β-Galactosidase Measurements

To monitor gene expression, the β-Galactosidase activity induced by reporter genes was measured using *o*-nitrophenyl-β-d-galactopyranoside [20,63]. After growth in liquid LB-MOPS-Carbenicillin (or LB-MOPS-tetracyclin for PAO1/P*_pelA_*-lacZ; antibiotics were used to avoid the growth of undesired strains) at 37 °C and 175 rpm for 18 h, *P. aeruginosa* PAO1 reporter strains were washed twice in fresh LB medium and resuspended in liquid LB-MOPS-Carbenicillin. *P. aeruginosa* PAO1 reporter strains inoculums (50 µL) were incubated (37 °C with 175 rpm agitation) for 18 h in 1 mL LB-MOPS-Carbenicillin (initial A_600nm_ of culture comprised between 0.020 and 0.025) supplemented with 10 µL of tested samples (including plant extracts, fractions of the ethyl acetate extract and purified compounds dissolved in DMSO) to reach a final concentration of 100 µg/mL or 10 µL of DMSO (1%, *v*/*v*). Additionally, naringenin or azithromycin, known as QS quenchers [18,64] were used as positive controls. After incubation, the bacterial density was assessed by spectrophotometry (A_600nm_) and the gene expression by the β-galactosidase assay.

### 4.6. Quantitative Analysis of Pyocyanin and Rhamnolipids Production

The production of pyocyanin was assessed according to previously described procedures [65,66]. *P. aeruginosa* PAO1 was grown for 18 h in liquid LB-MOPS. PAO1 cell suspension (50 µL) was added to 1 mL of LB-MOPS (starting A_600nm_ ranged between 0.02 and 0.025) supplemented with 10 µL of purified compounds dissolved in DMSO (100 µg/mL) or 10 µL of DMSO (1 %, *v*/*v*). After 18 h of growth, samples were taken to assess growth (A_600nm_) and pyocyanin production. Rhamnolipids were extracted and quantified by a methylene-blue-based method as described by Rasamiravaka et al. [67].

### 4.7. Biofilm Visualization and Quantification

*P. aeruginosa* PAO1 were grown overnight in LB medium at 37 °C with agitation and diluted with Biofilm Broth (BB) medium as described by Khalilzadeh et al. [68]; 25 µL of the diluted culture were added to 470 µL of BB medium (initial A_600nm_ of culture comprised between 0.14 and 0.16) supplemented with 5 µL of DMSO (1%, *v*/*v*) or plant extracts (100 µg/mL) or fractions of ethyl acetate extract (100 µg/mL) or purified compounds (from 6.25 to 800 µM) or oleanolic acid (800 µM) [19]. Depending on experiments, planktonic bacteria were discarded or transferred in sterile tube to assess the proportion of planktonic bacteria by colony forming units (CFU) measurement. The biofilms were washed three times with water (2 mL) and fixed with 2 mL of methanol (99%). After 15 min, the methanol was discarded, and the plates were dried at room temperature. Crystal violet (0.1% in water) was then added to each well (2 mL/well), and the plates were incubated for 30 min at room temperature. Crystal violet was then discarded, and stained biofilms were washed three times with 1 mL of water. Acetic acid (33% in water) was added to the stained biofilms (2 mL) in order to solubilize the crystal violet, and the absorbance of the solution was measured at 590 nm with a SpectraMax M2 device (Molecular Devices). To evaluate the impact of compounds on preformed biofilms, *P. aeruginosa* PAO1 cells were grown statically for 24 h to form biofilm; 1 mL of bacterial culture was then supplemented with 10 µL of DMSO (1%, *v*/*v*) or purified compounds (100 µM) for a further 24 h incubation and the biofilms were examined following the above procedure. Minimum biofilm inhibitory concentration 50% (MBIC_50_) of each compound and their eventual synergistic effects were investigated by using a checkerboard method [69] with concentrations from 6.25 to 800 µM. The Fractional Inhibitory Concentration Index (FICI) were calculated as follows: FICI = FICA + FICB; where FICA is the MBIC_50_ of drug A in combination/MBIC_50_ drug A alone and FICB is MBIC_50_ of drug B in combination/MBIC_50_ drug B alone. The interpretation was made as follows: Antagonistic (≥2), Indifferent (2 to 0.5), or Synergistic (≤0.5) [70]. All assays were performed in triplicate and repeated thrice.

The biofilm formation by *P. aeruginosa* PAO1 cells was also examined in glass coverslips cultures by fluorescence microscopy. The biofilm development and bacterial viability in biofilms were assessed using the LIVE/DEAD^®^ *Bac*Light™ bacterial viability kit (Invitrogen, Molecular probes). The growth medium was removed and replaced by 500 µL of a solution of SYTO 9 and propidium iodide diluted 400 fold in BB medium. Biofilms were incubated for 15 min and *P. aeruginosa* PAO1 cells were examined using a Leica DM IRE2 inverted fluorescence microscope coupled to a CCD camera (Leica DC350 FX, Leica Microsystems Inc., Bannockburn, IL, USA) and equipped with FITC and Texas red filters. Additionally, the antibiotic susceptibility of biofilm-encapsulated *P. aeruginosa* PAO1 cells was assessed using tobramycin as previously described [4]. Briefly, *P. aeruginosa* PAO1 cells were grown statically for 24 h to form biofilms, incubated with tobramycin (50 μg/mL = 107 µM) and DMSO (1%) or purified compounds (100 µM) for a further 24 h and assessed using the LIVE/DEAD *Bac*Light™ kit. To estimate the % viability of biofilm-encapsulated bacteria for each treatment, the glass coverslip was submerged in 2 mL of PBS solution and sonicated (WVR™ Ultrasonic cleaner, HF45KHz, 80W) for 1 min in order to unbind the biofilm. The collected biofilm suspension was adjusted to 0.5 A_600nm_ and then assessed for viability using the LIVE/DEAD^®^ *Bac*Light™ kit (Thermofisher Scientific, Waltham, MA, USA) according to the fluorescence microplate reader protocol described by the manufacturer.

### 4.8. Total Extracellular Polysaccharides and Alginate Quantification

Extracellular polysaccharides were extracted with ethanol and quantified with the phenol-sulfuric acid method described by Rasamiravaka et al. [4]. Alginate was extracted by cetyl pyridinium chloride precipitation and quantified by a modified carbazole-based method that detects uronic acids, as described by Rasamiravaka et al. [4].

### 4.9. Motility Assays

Swimming, swarming and twitching motilities were examined by using LB agar (0.3, 0.6 and 1%, respectively) as previously described by Rasamiravaka et al. [71] and Ha et al. [72]. After sterilizing and cooling (45–50 °C) LB agar, the test solutions were added (DMSO (1%; negative control) or purified compounds (100µM)), the medium was poured into compartmented Petri dishes and cooled to room temperature. Five microliters of bacterial culture (A_600_ = 1) were inoculated at the center of each compartment of the Petri dishes and incubated at 37 °C for 24 h (for swimming and swarming) or 48 h (for twitching motility). Bacteria spreading from the inoculation spot were measured with sliding caliper. For twitching motility, the agar was discarded from petri dish; twitching motility zones were visualized by staining for 1 min with 0.1% (*w*/*v*) of crystal violet as proposed by Darzins [73] and diameters measured.

## 5. Conclusions

The present study reports on the isolation, the identification and the characterization of anti-virulence properties of three terpenoids (cassipourol, β-sitosterol, α-amyrin) from *Platostoma rotundifolium*. Altogether, these non-microbicidal anti-virulence properties make terpenoids potential therapeutic agents against bacterial virulence, especially against major pathogenic bacteria such as *P. aeuginosa*, and support the wide anti-infectious use of *P. rotundifolium* in traditional Burundian medicine.

## Supplementary Materials

Supplementary materials can be found at www.mdpi.com/1422-0067/18/6/1270/s1.

## Abbreviations

AHLs	Acyl Homoserine Lactones
FICI	Fractional Inhibitory Concentration Index
MBC	Minimum Bactericidal Concentration
MIC	Minimum Inhibitory Concentration
QS	Quorum Sensing
