# Supplementary material for: Terpenoids from Platostoma rotundifolium (Briq.) A. J. Paton Alter the Expression of Quorum Sensing-Related Virulence Factors and the Formation of Biofilm in Pseudomonas aeruginosa PAO1
Source: Int J Mol Sci. 2017 Jun 14;18(6):1270. doi: 10.3390/ijms18061270 (PMC5486092; doi:10.3390/ijms18061270)
Supplement: Supplementary file 1 [file ijms-18-01270-s001.pdf]

Supplementary materials

Terpenoids from *Platostoma rotundifolium* (Briq.) A. J. Paton alter the expression of quorum sensing-related virulence factors and the formation of biofilm in *Pseudomonas aeruginosa* PAO1

Tsiry Rasamiravaka [1, 5, *, †], Jérémie Ngezahayo [2, 3, *, †], Laurent Pottier [2], Sofia Oliveira Ribeiro [2], Florence Souard [2, 6, 7], Léonard Hari [3], Caroline Stévigny [2], Mondher El Jaziri [1] and Pierre Duez [2, 4]

*Correspondence: (T Rasamiravaka), jrmienge2000@yahoo.fr (J Ngezahayo).

Supplementary Figures and Tables

1.1. Supplementary Tables

Table S1. *Pseudomonas aeruginosa* strains and plasmids used in this study.

Strains or plasmids	Relevant characteristics	References
P. aeruginosa PAO1	Wild-type (strain PAO0001; http://www.pseudomonas.med.ecu.edu/)	
P. aeruginosa PAO1/P_{pelA}-lacZ	PAO1 tagged by miniCTX-P_{pelA}-lacZ; Tc^r	[1]
pLP170	Broad-host-range *lac*Z transcriptional fusion vector containing an RNase III splice sequence positioned between the multiple cloning site and *lacZ*; Cb^r	[2]
pPCS1001	pLP170-derivative containing P_{lasR}-*lac*Z transcriptional fusion	[2]
pLPR1	pLP170-derivative containing P_{rhlI}-*lac*Z transcriptional fusion	[3]
pPCS1002	pLP170-derivative containing P_{rhlR}-*lac*Z transcriptional fusion	[2]
pLP170_*gacA*	pLP170- derivative containing P_{gacA}-*lac*Z transcriptional fusion	[4]
pLP170_*vfr*	pLP170- derivative containing P_{vfr}-*lac*Z transcriptional fusion	[4]
pQF50	Broad-host-range promoter-less *lac*Z transcriptional fusion vector; Cb^r	[5]
p 01	pQF50-derivative containing P_{lasB}-*lac*Z transcriptional fusion	[5]
p 02	pQF50-derivative containing P_{rhlA}-*lac*Z transcriptional fusion	[5]
p 03	pQF50-derivative containing P_{lasI}-*lac*Z transcriptional fusion	[5]
pTB4124	pQF50-derivative containing P_{aceA}-*lac*Z transcriptional fusion	[6]

Tc^r,tetracycline resistance; Cb^r carbenicillin resistance

Table S2: 600 MHz ^{1}H-NMR Spectral data for compounds **1-3** (δ ppm in chloroform-d_1; J Hz); bt: broad triplet; d = doublet; dd = doublet of doublets; m = multiplet; s = singlet; sp: septuplet; t = triplet

Position	Cassipourol (**1**)	β-sitosterol (**2**)	α-amyrin (**3**)
1	/	1.83 (m, 1H); 1.05 (m, 1H)	1.65 (m, 1H); 0.99 (m, 1H)
2	1.25 (m, 1H); 1.05 (m, 1H)	1.82 (m, 1H); 1.49 (m, 1H)	0.97 (m, 2H)
3	1.26 (m, 1H); 1.23 (m, 1H)	3.5 (m, 1H)	3.22 (dd, 1H, $^3J_{3\text{-}2}$=11.2, $^3J_{3\text{-}2}$=4.7)
4	1.12 (m, 2H)	2.27 (m, 1H) ; 2.21 (m, 1H)	/
5	1.50 (sp, 1H, ^3J=6.6)	/	0.74 (m, 1H)
6	1.25 (m, 1H); 1.05 (m, 1H)	5.33 (m, 1H)	1.52 (m, 1H); 1.39 (m, 1H)
7	1.25 (m, 1H); 1.05 (m, 1H)	1.97 (m, 1H); 1.94 (m, 1H)	1.54 (m, 1H); 1.35 (m, 1H)
8	1.28 (m, 1H); 1.23 (m, 1H)	1.42 (m, 1H)	/
9	1.36 (m, 1H)	0.91 (m, 1H)	1.52 (m, 1H)
10	1.25 (m, 1H); 1.07 (m, 1H)	/	/
11	1.40 (m, 2H)	1.47 (m, 1H); 1.45 (m, 1H)	1.91 (m, 2H)
12	1.97 (bt, 2H, $^3J_{11\text{-}12}$=7.7)	1.98 (m, 1H) ; 1.14 (m, 1H)	5.13 (t, 1H, $^3J_{11\text{-}12}$=3.6)
13	/	/	/
14	5.39 (m, 1H)	0.97 (m, 1H)	/
15	4.13 (d; 2H, $^3J_{14\text{-}15}$=6.9	1.56 (m, 1H); 1.05 (m, 1H)	1.25 (m, 2H)
16	0.85 (m, 3H)	1.82 (m, 1H); 1.24 (m, 1H)	1.6 (m, 2H)
17	0.82 (m, 3H)	1.09 (m, 1H)	/
18	0.84 (m, 3H)	0.66 (s, 3H)	1.31 (m, 1H)
19	0.83 (m, 3H)	0.98 (s, 3H)	0.91 (m, 1H)
20	1.65 (s, 3H)	1.34 (m, 1H)	1.31 (s, 1H)
21		0.9 (d, 1H, $^3J_{20\text{-}21}$=6.5	1.38 (m; 1H) ; 1.26 (s, 1H)
22		1.3 (m, 1H); 0.99 (m, 1H)	1.42 (m, 1H) ; 1.27 (m, 1H)
23		1.14 (m, 2H)	1.00 (s, 3H)
24		0.91 (m, 1H)	0.79 (m, 3H)
25		1.64 (m, 1H)	0.95 (s, 3H)
26		0.81 (m, 3H)	1.01 (s, 3H)
27		0.79 (s, 3H)	1.07 (s, 3H)
28		1.25 (m, 1H); 1.21 (m, 1H)	1.00 (s, 3H)
29		0.82 (m, 3H)	0.79 (m, 3H)
30			0.91 (s, 3H)

Table S3: 150 MHz ^{13}C-NMR Spectral data for compounds **1-3** (δ ppm in chloroform-d_1)

Position	Cassipourol (**1**)	β-sitosterol (**2**)	α-amyrin (**3**)
1	32.9	37.5	38.9
2	37.5	31.9	26.8
3	25.0	72.0	79.2
4	28.2	42.5	38.9
5	39.6	140.1	55.3
6	37.6	121.9	18.5
7	37.7	32.1	33.1
8	24.7	32.1	40.2
9	33.0	50.3	47.9
10	36.9	36.7	37.1
11	25.4	21.3	23.5
12	40.1	40.0	124.6
13	140.6	42.5	139.7
14	123.3	57.0	42.2
15	59.7	24.5	29.8
16	22.9	28.5	27.4
17	19.9	56.3	33.9
18	22.9	12.1	59.2
19	19.9	19.6	39.8
20	16.4	36.4	39.8
21		19.0	31.4
22		34.2	41.7
23		26.3	28.9
24		46.1	15.8
25		29.4	15.8
26		20.0	17.0
27		19.3	23.4
28		23.3	28.3
29		12.2	17.6
30			21.5

1.2. Supplementary Figures

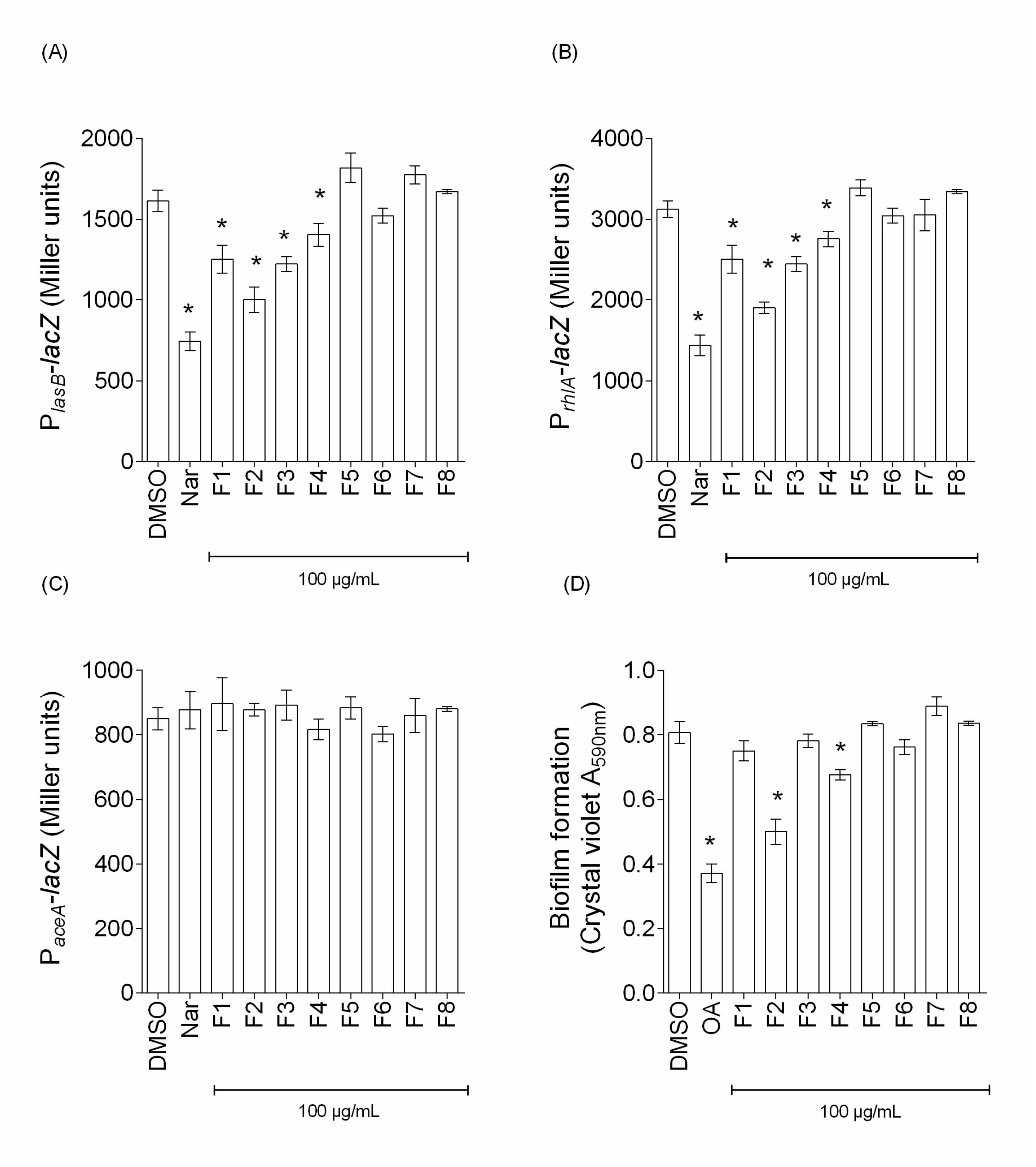

Figure S1. Effect of fractions from the EtOAc extract on the expression of QS-regulated (*lasB* (A), *rhlA* (B)) and QS-independent (*aceA* (C)) genes and on biofilm formation (D) in *P. aeruginosa* PAO1. Eight fractions (F1-F8) were tested at 100 µg/mL (for each fraction); Nar: naringenin (positive control for QS genes expression, 4 mM); OA: oleanolic acid (positive control for biofilm formation, 800 µM); DMSO: dimethylsulfoxide (negative control, 1% v/v). Gene expression was measured as the β-galactosidase activity of the *lacZ* gene fusions and expressed in Miller units and biofilm formation was quantified by crystal violet staining and measured at A_{590nm}. Error bars represent the standard errors of the means; all experiments were performed in quintuplicate with three independent assays and asterisks indicate samples that are significantly different from the DMSO (One-way ANOVA followed by Dunnett's test of multiple comparisons; $P < 0.01$).

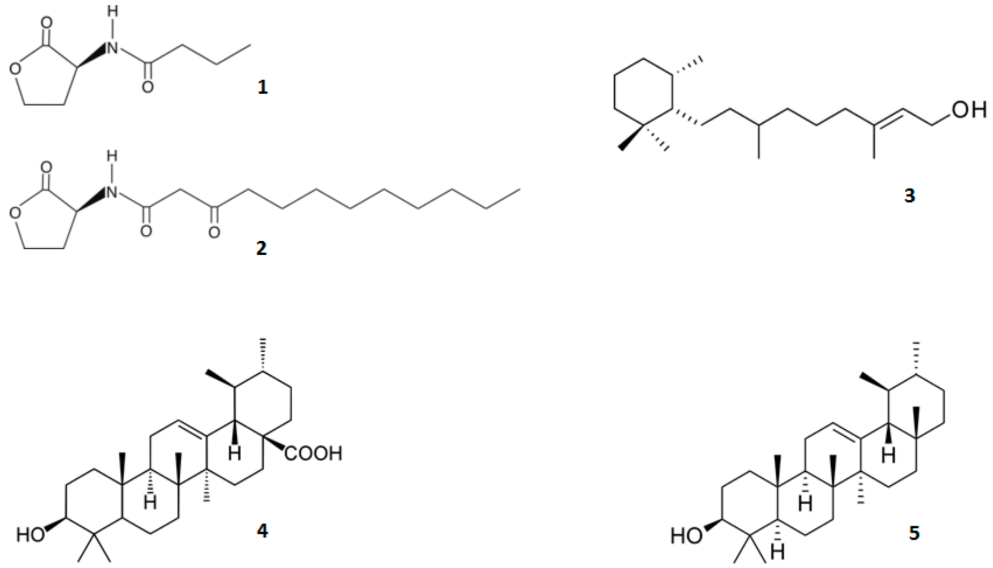

Figure S2. Structure of acylhomoserine lactones [*N*-(3-oxododecanoyl)-L-homoserine lactone (1) and *N*-butanoyl-L-homoserine lactone (2)], cassipourol (3), ursolic acid (4) and α-amyrin (5).

References

1. Chua, S.L.; Tan, S.Y.Y.; Rybtke, M.T.; Chen, Y.; Rice, S.A.; Kjelleberg, S.; Tolker-Nielsen, T.; Yang, L.; Givskov, M. Bis-(3′-5′)-cyclic dimeric GMP regulates antimicrobial peptide resistance in *Pseudomonas aeruginosa*. *Antimicrob. Agents Chemother.* **2013**, *57*, 2066–2075.
2. Pesci, E.C.; Pearson, J.P.; Seed, P.C.; Iglewski, B.H. Regulation of *las* and *rhl* quorum sensing in *Pseudomonas aeruginosa. J. Bacteriol.* **1997**, *179*, 3127–3132.
3. Van Delden, C.; Iglewski, B.H. Cell-to-cell signaling and *Pseudomonas aeruginosa* infections. *Emerg. Infect. Dis.* **1998**, *4*, 551–560.
4. Rasamiravaka, T.; Vandeputte, O.M.; Pottier, L.; Huet, J.; Rabemanantsoa, C.; Kiendrebeogo, M.; Stévigny, C.; Duez, P.; El Jaziri, M. *Pseudomonas aeruginosa* biofilm formation and persistence, along with the production of quorum sensing dependent virulence factors, are disrupted by a triterpenoid coumarate ester isolated from *Dalbergia trichocarpa*, a tropical "legume". *PLoS ONE* **2015**, *10*, e0132791.
5. Ishida, T.; Ikeda, T.; Takiguchi, N.; Kuroda, A.; Ohtake, H.; Kato, J. Inhibition of quorum sensing *Pseudomonas aeruginosa* by *N*-acyl cyclopentylamides. *Appl. Environ. Microbiol.* **2007**, *73*, 3183–3188.
6. Kretzschmar, U.; Khodaverdi, V.; Jeoung, J.H.; Görisch, H. Function and transcriptional regulation of the isocitrate lyase in *Pseudomonas aeruginosa*. *Arch. Microbiol.* **2008**, *190*, 151–158.
